# A Terrible Future: Episodic Future Thinking and the Perceived Risk of Terrorism

**DOI:** 10.3389/fpsyg.2019.02333

**Published:** 2019-10-22

**Authors:** Simen Bø, Katharina Wolff

**Affiliations:** Department of Psychosocial Science, Faculty of Psychology, University of Bergen, Bergen, Norway

**Keywords:** episodic future thinking, episodic foresight, future thinking, risk perception, terror risk, perceived risk, tourism, open science

## Abstract

Terrorism is a salient risk source in 21st century life and may deter tourists from visiting certain destinations. How people perceive the risk of a future terror attack abroad, and thus their traveling decisions, may be influenced by whether they think about the future in specific and personal terms (episodic future thinking) or in more general, abstract terms (semantic future thinking). In a pre-registered experiment (*N* = 277) we explored the potential impact of episodic future thinking on the perceived risk of terror attacks abroad. Participants were randomly assigned to one of four conditions: (1) An episodic future thinking-condition, where participants were asked to imagine a specific, terror-related personal episode that might occur in the future while traveling abroad; (2) a semantic future thinking-condition, where participants were asked to think more abstractly about terror events that might occur in the future; (3) an episodic counterfactual thinking-condition, where participants were asked to imagine a specific, terror-related personal episode that might have occurred in the past while traveling abroad and (4) a passive control condition. Participants indicated their perceived risk of six different future terror attacks occurring abroad. The manipulation checks suggest that the experimental manipulations functioned as intended. Contrary to the central hypothesis of the study, there were no differences in the perceived risk of terror attacks between the conditions. These results run counter to previous research and do not support the idea that how people think about the future influences their perceived risk of future dramatic events. Potential limitations and implications are discussed.

## Introduction

Terrorism is a salient threat in modern life. Although recent years has seen a decrease in the number of people killed in terror attacks, there has been an increase in the number of civilians killed and the number of countries affected by terrorism (Institute for Economics and Peace [IEP], 2018). Realistically, the most fatal terrorist attacks occur in a limited number of countries, most of which are active conflict zones. However, there have also been several terror attacks targeting tourism hotspots in the last few years, including an armed assault at a nightclub in Istanbul (Turkey), a suicide bombing at a concert in Manchester (United Kingdom), and a van attack in Barcelona (Spain; Institute for Economics and Peace [IEP], 2018).

Research suggests that tourists worry little about terror attacks and do not perceive them as very risky compared to other hazards (i.e., Wolff and Larsen, 2016, 2017). However, several studies suggest that the perceived risk of terrorism may act as a travel deterrent, meaning that how people perceive terror events is important to consider if we wish to understand their decision making (Sönmez and Graefe, 1998a, b; Gray and Wilson, 2009; Larsen, 2011). Psychological research may inform our understanding of how people are affected by terrorism, particularly the psychological processes involved in individuals’ perception of past and future terror attacks, and the cognitive, affective, and behavioral effects of these perceptions.

*Perceived risk* describes our perception of the probability of negative outcomes weighted by their magnitude. In other words, perceived risk has both an uncertainty aspect (the probability of the future event) and a severity aspect (the magnitude of the future event; Brun, 1994; Wolff and Larsen, 2017). Some studies indicate that different hazards may act as deterrents to travel, and several studies suggest an association between risk perception and travel intentions, in the sense that people generally have weaker intentions to visit destinations that are perceived as riskier (Sönmez and Graefe, 1998a, b; Gray and Wilson, 2009). Studies on related concepts such as perceived threat suggest that the relationship between perceived risk and behavior might depend on whether risk is assessed as the risk to oneself or the general/national risk, with a stronger association for perceived personal risk (Huddy et al., 2002; Goodwin et al., 2005). Although some studies have found no relationship between the perceived risk of terrorism and intentions to travel (i.e., Floyd et al., 2004), the overall trend seems to suggest that risk perception may influence decisions.

Risk perception is also an important driving force in the social and economic consequences of risk events. Based on the social amplification of risk framework (Kasperson et al., 1988), it can be argued that a collective increase in perceived risk can result in severe secondary impacts, for example in terms of negatively impacting the local tourism industry. The main idea is that risk perception functions as the mechanism through which a risk event has social and economic consequences. In this description, risk perception functions as one of several signals containing information about the risk event and serves as an antecedent to risk-related behavior, culminating in widespread secondary consequences (Kasperson et al., 1988). This, in combination with the aforementioned implications for travel decisions, solidifies the importance of understanding the influences on how people perceive terrorism risk and suggests that exploring potential antecedents to perceived risk is important to enhance our understanding of psychological factors influencing tourists’ travel decisions and behavior.

Several studies have explored psychological antecedents to risk perception. For example, it seems that people’s recollection of prior risk estimates can be influenced by their current beliefs, so they remember seeing the world as safer than they actually did (Fischhoff et al., 2005). Other predictors of perceived risk include certain individual characteristics, such as openness to change and hedonism, where greater openness is associated with reduced perceived risk and greater hedonism with increased perceived risk. Additional predictors include situational characteristics such as the way that information is framed, where some frames are assumed to be risk attenuating (Kapuściński and Richards, 2016). Experimental studies on risk perception, such as studies on the impact of news framing on perceived risk for terror attacks (Kapuściński and Richards, 2016), and studies exploring how characteristics of terror attacks influence perceived risk (e.g., Wolff and Larsen, 2017), are necessary to establish causal relationships between predictors of risk perception and risk perception.

One potential predictor of perceived risk that can be manipulated experimentally is *episodic future thinking* (EFT). Foremost, EFT is a form of future-oriented cognition that concerns specific, plausible, personal episodes (Atance and O’Neill, 2001). This differs from *semantic future thinking* (SFT), which is future-oriented and may be personal, but which concerns a general, non-specific future (Abraham et al., 2008; see Szpunar et al., 2014, for a taxonomy of future-oriented cognition). Just as our episodic memory allows us to re-experience our past, EFT allows us to pre-experience a specific, personal event before it occurs (Atance and O’Neill, 2001; Szpunar, 2010). For example, a person can remember what it was like to stroll the beaches of Sicily last summer, and also imagine what it will be like to go swimming in Crete next year.

We mentally construct our own futures using both episodic and semantic memories, an ability that confers a tremendous adaptive advantage, particularly by strengthening our decision-making capabilities (Suddendorf and Corballis, 1997; Addis et al., 2007; Klein, 2013). Envisioning future episodes evidently makes it possible to alleviate *delay discounting*, the general tendency to prioritize immediate, smaller rewards over future, larger rewards (Daniel et al., 2015; Bulley et al., 2016; Stein et al., 2018). For example, if people tend to prioritize spending money immediately as opposed to saving it for a vacation, EFT about said vacation might strengthen their ability to take future outcomes into account, and thus enhance long-term decision making. Much of the research on EFT is centered around this relationship with intertemporal decision making (Miloyan and McFarlane, 2019). Furthermore, a large number of the studies on EFT have emphasized simulations of positive future episodes (see Calluso et al., 2019, for a notable exception). However, EFT might also be important for decision making in other areas, and through other mechanisms, particularly when imagining potential negative future episodes.

While vividly imagining positive future episodes might allow us to prioritize bigger, delayed rewards over smaller, immediate rewards, imagining negative personal futures might help us prepare for potential adverse outcomes (Miloyan et al., 2014, 2016). For example, people planning their next holiday trip might not only see themselves Scooba diving or hiking, they may also imagine themselves experiencing a natural disaster or a terror attack, and thus perceive the risk of this hazard as greater than they would otherwise. This would probably impact the person’s intentions to travel and potentially their actual travel decisions. Based on several studies suggesting that people frequently engage in EFT in daily life (D’Argembeau et al., 2011; Barsics et al., 2016), it is reasonable to assume that they may also think about future, personal episodes abroad when making vacation choices. There are both empirical and theoretical reasons for assuming that EFT may be associated with increased travel-related risk perceptions, which would be one way in which this form of future thinking influences tourists’ decision making.

Some speculate that EFT influences our decisions through providing information about the affective desirability of future outcomes and the possibility that these outcomes may occur (Bulley et al., 2016). Specifically, a person imagining a potential future event (like enjoying a forthcoming vacation or experiencing a terror attack in a foreign country) will anticipate certain emotions if this event comes to fruition and will also evaluate the event for its likelihood of occurrence. Supporting this, some studies suggest that imagining personal future episodes multiple times may heighten the perceived probability that the imagined events will occur (Szpunar and Schacter, 2013). One possibility is that EFT can influence decisions in a broader way than previously considered, by not only providing information about affective desirability, but providing information about the severity of future outcomes on a broader scale. If so, there are grounds for assuming that EFT might also guide our decisions through affecting how we perceive risk. By mentally experiencing a future threat, we may be more convinced about its severity and probability of occurrence, and thus perceive the threat as riskier. Thus, we argue that thinking about personal exposure to future terror attacks may increase how risky people perceive such attacks to be. Several mechanisms explaining this phenomenon are conceivable.

One mechanism that could explain why EFT might increase risk perception is the fact that EFT more than SFT may increase availability, i.e., the ease and vividness with which an event comes to mind. It is well known that events that come to mind easily (high availability) and with great vividness are judged more likely and hence more risky (Slovic et al., 1981; Visschers et al., 2012). Therefore, a person having a vivid imagination of a potential future event might judge the risk of the event occurring as higher because of the increased availability. For example, a tourist vividly imagining experiencing a possible natural disaster or act of terror might have this more available when making judgments about the risk of such an event, and thus perceive the risk as greater than they would otherwise. As EFT is defined through an increased specificity and vividness, it is conceivable that this form of future thinking will lead people to perceive the risk of the event as greater than the more abstract and general way of thinking about the future that characterizes SFT.

Another explanation could be that EFT decreases the *psychological distance* with which people view future terror attacks. Psychological distance describes the subjective, perceived remoteness of an event, and is commonly understood as consisting of four domains: spatial distance, temporal distance, social distance and hypotheticality (Trope and Liberman, 2010; Zwickle and Wilson, 2013). A number of studies suggest that EFT helps us consider consequences which are psychologically distant (Daniel et al., 2015; Bulley et al., 2016; Stein et al., 2018). Furthermore, psychological distance is related to risk perception, in the sense that events perceived as psychologically proximal are also perceived as riskier (Zwickle and Wilson, 2013). If people view terror attacks as psychologically distant, for example in terms of spatial distance, EFT could possibly decrease the perceived distance and thus increase the perceived risk. For example, potential tourists could perceive acts of terror as spatially distant when planning their vacation but imagining personally experiencing a terror attack in the future might reduce this perceived distance, and thus increase perceived risk.

A third rationale for assuming that EFT will increase perceived risk compared to SFT is the importance of personal experience in explaining perceived risk (Weber, 2006; Van der Linden, 2015). Several studies suggest that recent experience with a hazard increases the perceived risk of this hazard, usually by reducing the psychological distance with which this hazard is perceived. EFT may serve as a substitute for personal experience in a way in which SFT does not, and episodic simulations may be attributed approximately similar evidentiary value as actual experience (Kappes and Morewedge, 2016). Assuming that EFT may function as a substitute for personal experience in impacting decision making, there are clear reasons for assuming that EFT will heighten perceived risk as compared to SFT. Evidently, there are several highly plausible explanations for why there may be a connection between engaging in EFT and perceiving the risk of a future outcome as higher. As discussed above these include increased vividness and availability for the event, decreased psychological distance to the event, and vicariously experiencing the event.

Some prior studies have been conducted on future-oriented imagination and perceived probability (i.e., Sherman et al., 1985), finding that outcomes which are easy to imagine are also perceived as more probable; however, the future thinking has not been specific in place or in time, and the studies have typically focused on perceived probability as opposed to perceived risk. Some more recent studies have highlighted relationships between EFT and perceived probability (Szpunar and Schacter, 2013), and the implications of EFT on risk taking (e.g., Bulley et al., 2019). However, there are a very limited number of studies on the association between the simulation of specific, future, personal episodes and risk perception.

We are aware of only one study exploring the implications of EFT on how people perceive the risk of hazards (Lee et al., 2018). Lee and colleagues explored the possibility that EFT might impact the perceived risk of environmental challenges, and thus also have an impact on environmental behavior. EFT about possible future environmental challenges was found to predict environmentally friendly behavior, an effect that was mediated by the perceived risk of these challenges. However, the authors operationalized perceived risk using a measure of perceived probability, and it is not clear whether this effect extends to risk perception, as lay peoples’ risk perceptions suffer from probability neglect. This means that they almost exclusively rely on severity and ignore probability when judging risk (Sunstein, 2002; Slovic and Peters, 2006). Furthermore, it is an open question whether EFT would have a comparable impact on the perceived risk of future terror attacks as environmental challenges, as these are different hazards, necessitating further empirical research.

Based on an assumption that EFT might be consequential for perceived risk, we argue that whether people think about the possibility of future terrorism in episodic or semantic terms might impact how they perceive the risk of terrorism. To our knowledge, this issue has not been explored in previously published research, and seeing as risk perception may impact tourist decisions, exploring such research questions may have important implications for understanding human behavior. This is also a good example of how concepts which are important in mainstream psychology, specifically EFT, can be applied in investigating a tourist-relevant concept, in this case how the perceived risk of terror attacks occurring abroad may influence tourists’ travel decisions. Specifically, we conducted an experimental study exploring whether participants thinking about the future episodically perceived the risk of future terror attacks on vacation as greater than participants thinking about the future semantically.

## Materials and Methods

### Overview and Hypotheses

To assess whether episodic future thinking can increase the perceived risk of future terror attacks, we conducted a pre-registered experimental study with four conditions: (1) an episodic future thinking-condition (EFT-condition), (2) a semantic future thinking-condition (SFT-condition), (3) an episodic counterfactual thinking-condition (ECT-condition) and (4) a passive control condition. In the EFT-condition participants were asked to imagine a specific, personal episode in the future. The episodic counterfactual-thinking condition had identical instructions, with the exception that participants were asked to imagine that the event could have taken place in the past, based on a memory of sitting in a restaurant while abroad. Participants in the SFT-condition were asked to think about the future in an abstract, impersonal way. Specifically, participants were asked to think about three examples of terror events that could occur abroad in the future.

The main argument for using an additional episodic condition to the EFT-condition is that it helps establish whether the effect is specific to episodic *future* thinking or is an effect of episodic thinking more generally (Hollis-Hansen et al., 2019). As there is reason to believe that participants have not experienced comparable personal, past events to an imagined, future terror attack, *episodic counterfactual thinking* (ECT) was deemed a suitable condition to explore whether the potential effect of EFT was isolated to EFT or generalizable to other forms of episodic thinking. ECT concerns specific episodes which did not, but could have, occurred in the past (Epstude and Roese, 2008; De Brigard and Parikh, 2019). Similar to the distinction between EFT and SFT, it is possible to distinguish between episodic and semantic *counterfactual thinking* (De Brigard and Parikh, 2019). Imagining what would have happened if you had missed your flight would be an example of ECT, whereas imagining what the world would be like without cruise ships would be an example of semantic counterfactual thinking.

The experiment was conducted as an online survey experiment using Qualtrics. The study was pre-registered prior to data collection (online pre-registration at https://osf.io/f4s9r). Full descriptions of the instructions in the experimental conditions, the procedure used in the study and a summary of the deviations from the pre-registration are available at https://osf.io/ygptx/?view_only=7861eaaf164646d0a4682d5018c3d38. The data are available at the aforementioned webpage, and as Supplementary Data Sheet S1.

Hypotheses for this study pertained both to the manipulation checks and the effect of future thinking on perceived risk.

Hypotheses for the manipulation checks: (1) Participants assigned to both the EFT-condition and the ECT-condition will have a higher mean score than participants assigned to the SFT-condition on the index of vividness, the index of visual perspective and the item measuring feeling of mentally experiencing the imagined event. (2) Participants in the EFT-condition and the SFT-condition will have mean scores above the midpoint (4.0) on the item measuring the degree of future thinking. Similarly, participants in the ECT-condition will have mean scores above the midpoint (4.0) on the item measuring the degree of counterfactual thinking.

Main hypothesis: Participants assigned to an EFT-condition will have a higher mean rank value on the index of perceived risk of terror attacks than participants assigned to an ECT-condition, an SFT-condition and a passive control condition.

### Sample and Procedure

#### Sample Size Calculation

We used G^∗^Power (Version 3.1.9.4) to conduct a power analysis to calculate sample size for the analyses testing the main hypotheses (Mann–Whitney U *post hoc* tests, described in the analysis section). The expected effect size is based on effects found in prior research (e.g., Wolff and Larsen, 2017; Lee et al., 2018; effect size *d* = 0.5, alpha level = 0.0167, power = 0.80, logistic distribution), suggesting a minimal sample size of 264 to achieve sufficient power. Although there is a limited number of prior studies, making the estimate vulnerable to influence from sampling error (Schäfer and Schwarz, 2019), several studies using EFT as the independent variable have found effects larger than the conventional medium effect of 0.5 (see Hollis-Hansen et al., 2019, for a mini-review). This suggests that there are ample grounds for assuming an effect of at least a medium size. To compensate for some potential participants dropping out of the study we aimed to recruit 275 participants.

#### Sample

Two hundred and seventy seven participants (63 men, 214 women) were recruited using the Citizen lab administered by the Digital Social Science Core Facility, University of Bergen, and through recruitment of students at the Faculty of Psychology, University of Bergen. Students at the Faculty of Psychology were recruited using flyers at the campus, and through recruitment in lectures. We deviated from the pre-registration in this respect, as we were unable to recruit a sufficient number of participants through the Citizen lab, which necessitated recruitment of additional participants through other means. Participants had an average of 2.58 years of higher education (*SD* = 2.15). Age was not registered in order to secure the anonymity of all participants (mentioned in the ethics statement below).

#### Procedure

Participants were randomly assigned to conditions with simple randomization using the randomizer function in Qualtrics. This means that every participant had an equal chance (25%) to be assigned to one of four blocks, each block representing one of the four conditions.

Participants arrived at the Citizen Lab or at a data lab at the Faculty of Psychology and were informed that the study they were about to participate in focused on how people think about terror attacks. Thereafter, they were seated at individual desks. They read an introductory statement for the study and indicated their informed consent. Following this, they were asked to indicate their gender and how many years of higher education they had. Subsequently, the participants in the three experimental conditions were exposed to the experimental induction, whereas participants in the control condition (*n* = 55) only answered questions pertaining to their perceived risk of terror.

In the EFT-condition (*n* = 65), participants were asked to imagine a specific, personal episode in the future. The instructions asked participants to vividly imagine that they are sitting in a restaurant while on a vacation abroad, and that they suddenly hear explosions and shooting close by. Thereafter, they were asked to imagine further development of the dramatic situation. The episodic counterfactual-thinking condition (*n* = 77) had identical instructions, with the exception that participants were asked to imagine that the event could have taken place in the past, based on a memory of sitting in a restaurant while on vacation abroad. Participants in the SFT-condition (*n* = 80) were asked to think about the future in an abstract, impersonal way. Specifically, participants were asked to think about three examples of terror events that could occur abroad in the future. The specific scenarios in the EFT-condition and the ECT-condition were based on the most frequent terror attacks in the last 20 years reported in the Global Terrorism Index (Institute for Economics and Peace [IEP], 2018). A full description of the wording used in the experimental manipulations can be found in the OSF-folder for this study^1^.

After the experimental manipulation, participants answered the manipulation checks and the questions measuring potential covariates. Thereafter, they answered questions pertaining to their perceived risk of terror, before finally being given a written debrief describing the purposes of the study in full.

### Measures

#### Demographic Measures

Gender was measured with three categories (1 = male, 2 = female, 3 = do not wish to report gender). Years of education was measured using one item, *How many years of higher education have you completed?*

#### Manipulation Checks

As per the norm in the field we included phenomenological measures to assess participants’ experience of their thought content (Miloyan and McFarlane, 2019). A strong argument for using phenomenological, self-report measures as manipulation checks on participants’ thinking is that participants may have better access to their own mental processes than external observers (Miloyan and McFarlane, 2019; Miloyan et al., 2019).

An index of vividness was constructed from the following three 7-point Likert-type items, with endpoints 1 (completely disagree) and 7 (completely agree): *I imagined one specific event*, *my thoughts were vivid*, *my thoughts were concrete*. The scores on the index had moderately satisfactory internal consistency (α = 0.73).

Counterfactual/future thinking was measured using one item on a scale from 1 to 7, with endpoints 1 (completely disagree) and 7 (completely agree). EFT and SFT-condition: *I thought about the future*. ECT-condition: *I thought about an alternative to something that happened in the past*.

Feeling of mentally experiencing the event(s) was measured with one item on a scale from 1 and 7, with endpoints 1 (completely disagree) and 7 (completely agree). EFT- and SFT-condition: *I was taken forward in time to when the event(s) might take place*. ECT-condition: *I was taken back in time to when the event could have taken place.*

Visual perspective was measured using the following three 7-point Likert-type items, with endpoints 1 (completely disagree) and 7 (completely agree): *I experienced the event as if I observed myself from the outside* (reversed), *I experienced the event through my own eyes*, *I experienced the event as if I was not present myself* (reversed). In the SFT-condition, visual perspective was measured with the same three items, using the word “events” instead of “event.” We had a pre-registered plan to construct an index of visual perspective using these three items. However, the scores had unsatisfactory internal consistency (α = 0.57). Thus, differing from the pre-registration, field perspective and observer perspective were analyzed separately, and we did not analyze the third item.

#### Covariates

The following concepts were measured using one-item scales with endpoints 1 (completely disagree) and 7 (completely agree). Judged realism: *the imagination of the event was realistic*. Subjective difficulty of generating a scenario: *it was easy to perform the task where I was asked to imagine an event(s)*. Perceived temporal distance: *when you imagined the event(s), how far away in time did you perceive it(them) to be?* Time spent on imagining either future episodes or counterfactual episodes or thinking about future semantic events, was measured in seconds using a timing question in Qualtrics.

#### Dependent Variable

It is important, when exploring potential antecedents of perceived risk, to operationalize the concept in a way that reflects our conceptual understanding of the components of risk perception, seeing as certain factors may influence perceived probability or perceived severity selectively. We measured the perceived risk of terror using an index composed of six items for different forms of terror. For all items, participants were asked to indicate their perceived risk of terror attacks abroad. Only the index was used for the pre-registered analyses. Specifically, participants were asked the following questions: *How do you perceive the risk of a bomb explosion at a public place/a biological terrorist attack/a chemical terrorist attack/a terror-related car attack/an armed terrorist attack/the hijacking of a flight*? All items were on a scale from 1 to 7, with endpoints 1 (not risky) and 7 (very risky). The scores on the index had excellent internal consistency (α = 0.89). For terrorism in general, participants were asked how they perceive the risk of being exposed to terrorism, on a scale from 1 to 7 with endpoints 1 (not risky) and 7 (very risky). All alpha values mentioned in this methods section were interpreted in relation to Ponterotto and Ruckdeschel (2007).

### Ethics Statement

This work complied with the general guidelines for research ethics by the Norwegian National Committees for Research Ethics in the Social Sciences and the Humanities (NESH). The data were not covered by the Norwegian Personal Data Act, and thus this project was exempt from submitting a formal application to the Data Protection Official for Research. Also, as the study did not include research questions related to health, there was no requirement to formally apply to the Regional Committee for Medical and Health Research Ethics, as the study was not covered by the Norwegian Health Research Act. Participants marked their informed consent before participation, in accordance with the Declaration of Helsinki (World Medical Association, 2013). As age could be a potential identifier for the participants in the Citizen lab administered by the Digital Social Science Core Facility, the age of participants was not included as a demographic question. The participants received a written debrief explaining the purposes of the study after completing the questionnaire^2^.

## Results

### Analysis Plan

All analyses were run using IBM SPSS [Version 25]. For several of the analyses involving the index of perceived risk, our pre-registration notes that we planned to conduct non-parametric tests. Specifically, we planned to conduct a Kruskal–Wallis omnibus test for overall group differences, with Mann–Whitney *U post hoc* tests. We also planned to analyze the relationship between the potential covariates and risk perception using Spearman correlation coefficients. This was based on prior research and pilot testing suggesting that the scores would be highly skewed. However, the distribution was a much closer approximation to a normal distribution than we thought it would be. As such, we opted for the more powerful parametric tests (specifically, Pearson correlation as opposed to Spearman correlation for some of the manipulation check analyses, and one-way ANOVA as opposed to Kruskal–Wallis for the main analysis).

To analyze differences on the manipulation check items in the three experimental conditions, we ran three one-way between-subject ANOVAs. Any significant differences were followed up with independent *t*-tests. For the items measuring future thinking and counterfactual thinking, we used three one-sample *t*-tests to compare the obtained scores in the EFT-condition, the ECT-condition and the SFT-condition with the scale midpoint (4.0).

To explore relationships between the potential covariates and the dependent variable (i.e., terror risk perceptions), we computed Pearson coefficients between scores on each of the four potential covariates (time spent on the task, judged realism, subjective difficulty of generating a scenario, and perceived temporal distance) and scores on the index of perceived risk. For the main analysis (the effect of experimental condition on perceived risk), we ran a one-way between-subjects ANOVA with experimental condition as the between-subjects factor.

### Data Exclusion and Missing Values

Outliers were defined using the MAD-median rule (Wilcox, 2012). Outliers were assessed separately for each condition, as grouped data were used in the analyses (Tabachnick and Fidell, 2014). Unless otherwise reported, there were no outliers for the variables used in the analyses. Where there were outliers, these were retained in analyses, and all the analyses involving scores with outliers were run both with and without the outliers (Howitt and Cramer, 2011). Any differences in results are noted. As there were no missing data we excluded cases analysis-by-analysis in SPSS (Tabachnick and Fidell, 2014).

### Main Results

#### Preliminary Analyses

The distribution of gender was not significantly different depending on condition, χ2(1,*N* = 277) = 1.86, *p* = 0.60, φ = 0.08. The scores on the higher education variable were not significantly different depending on condition, *F*_3__,__274_ = 0.12, *p* = 0.95, η^2^ = 0.001. This did not change when outliers were excluded, *F*_3__,__251_ = 0.66, *p* = 0.58, η^2^ = 0.008. Thus, conditions were comparable on demographic variables.

#### Manipulation Checks

As predicted, there was a significant difference on the index of vividness depending on condition, *F*_2__,__220_ = 29.82, *p* < 0.001, η^2^ = 0.22. Participants in the EFT-condition (*M* = 5.56, *SD* = 1.00) and the ECT-condition (*M* = 5.65, *SD* = 0.90) reported a significantly higher degree of vividness than participants in the SFT-condition (*M* = 4.49, *SD* = 1.19). Both comparisons showed an estimated large effect size (Table 1). Participants in the EFT-condition did not have a significantly different vividness score from participants in the ECT-condition.

**TABLE 1:** Independent *t*-tests comparing scores on phenomenological measures in the three experimental conditions.

**Measure**	***t***	**df**	***p***	***d***	**95% CI**
**EFT vs. SFT**					
Vividness	5.80	143	<0.001	0.97	[0.70, 1.44]
Mentally experiencing the event	2.14	143	0.03	0.36	[0.05, 1.17]
Field perspective	3.70^a^	142.62	<0.001	0.61	[0.58, 1.91]
**ECT vs. SFT**					
Vividness	6.88	155	<0.001	1.10	[0.83, 1.50]
Mentally experiencing the event	4.18	155	<0.001	0.66	[0.60, 1.69]
Field perspective	4.22	155	<0.001	0.67	[0.75, 2.07]
**EFT vs. ECT**					
Vividness	−0.56	140	0.58	0.10	[−0.40, 0.23]
Mentally experiencing the event	−1.93	140	0.06	−0.32	[−1.09, 0.13]
Field perspective	0.50	140	0.62	−0.08	[−0.81, 0.48]

*EFT, episodic future thinking; SFT, semantic future thinking; ECT, episodic counterfactual thinking. Using a Bonferroni correction for multiple comparisons, the alpha-level was adjusted to 0.0167. ^*a*^Corrected *t*-value (the assumption of equal variances was violated).*

As predicted, there was a significant difference on the feeling of mentally experiencing the event depending on condition, *F*_2__,__220_ = 9.02, *p* < 0.001, η^2^ = 0.08. This did not change when outliers were excluded, *F*_2__,__211_ = 27.50, *p* < 0.001, η^2^ = 0.21. Participants in the EFT-condition (*M* = 4.65, *SD* = 1.62) and participants in the ECT-condition (*M* = 5.18, *SD* = 1.67) reported mentally experiencing the event to a significantly higher degree than participants in the SFT-condition (*M* = 4.04, *SD* = 1.76). However, controlling for an increased probability of type 1 error with multiple comparisons using a non-registered Bonferroni correction implies adjusting the alpha value from 0.05 to 0.0167. While significant with the uncorrected alpha value, the difference between the EFT-condition and the SFT-condition was no longer significant at the corrected alpha value (*p* = 0.03). Participants in the EFT-condition did not have a significantly different feeling of mentally experiencing the event than participants in the ECT-condition (Table 1).

As scores on the index of visual perspective had lower internal consistency than expected, we deviated from the pre-registered analysis plan by analyzing field perspective and observer perspective separately. Contrary to our predictions, there was no significant difference in observer perspective depending on condition, *F*_2__,__219_ = 0.24, *p* = 0.79, η^2^ = 0.002. As predicted, there was a significant difference in field perspective depending on condition, *F*_2__,__219_ = 11.20, *p* < 0.001, η^2^ = 0.09. Participants in the EFT-condition (*M* = 5.03, *SD* = 1.86) and participants in the ECT-condition (*M* = 5.19, *SD* = 1.99) reported a significantly higher degree of field perspective than participants in the SFT-condition (*M* = 3.79, *SD* = 2.18). Both comparisons showed an estimated medium-to-large effect size (Table 1). Participants in the EFT-condition did not have a significantly different degree of field perspective than participants in the ECT-condition.

Three one-sample *t*-tests were run to explore the degree of future/counterfactual thinking in the three experimental conditions. Participants in the EFT-condition (*M* = 4.91, *SD* = 1.84), the ECT-condition (*M* = 5.16, *SD* = 1.95) and the SFT-condition (*M* = 5.26, *SD* = 1.51) had higher scores than the scale midpoint of 4.0 (Table 2), suggesting that participants were engaged in future thinking in the EFT- and SFT-conditions and counterfactual thinking in the ECT-condition. The effect sizes were medium-to-large (Table 2). These results were still significant when outliers were excluded (Table 2).

**TABLE 2:** One sample *t*-tests for scores on future/counterfactual thinking.

**Condition**	**Test score**	***t***	**df**	***p***	***d***	**95% CI**
**With Outliers Included**						
EFT	4	3.97	64	<0.001	0.49	[0.45, 1.36]
ECT	4	5.21	76	<0.001	0.59	[0.71, 1.60]
SFT	4	7.94	79	<0.001	0.83	[0.93, 1.60]
**Without Outliers**						
EFT	4	4.28	62	<0.001	0.60	[0.60, 1.47]
ECT	4	9.69	68	<0.001	1.17	[1.30, 1.97]
SFT	4	8.14	78	<0.001	0.92	[0.99, 1.64]

*EFT, episodic future thinking; SFT, semantic future thinking; ECT, episodic counterfactual thinking.*

#### Covariates

Perceived risk was not significantly associated with judged realism (*r* = 0.05, *N* = 222, *p* = 0.45), subjective difficulty of generating a scenario (*r* = 0.04, *N* = 222, *p* = 0.60), perceived temporal distance (*r* = 0.02, *N* = 222, *p* = 0.79) or the amount of time spent on the task (*r* = −0.13, *N* = 222, *p* = 0.06). This did not change when outliers were excluded (Table 3). As none of the measured potential covariates were associated with perceived risk, none were included in the analyses for the effect of future thinking on perceived risk.

**TABLE 3:** Pearson correlations between risk perception and covariates without outliers.

		**Judged realism**	**Subjective difficulty of generating a scenario**	**Perceived temporal distance**	**Amount of time spent on the task**
	*r*	0.04	0.007	0.03	−0.10
Perceived risk	*p*	0.14	0.92	0.67	0.14
	*N*	206	213	219	206

#### Effect of Episodic Future Thinking on Perceived Risk

As the scores on the dependent variable were more normally distributed than expected, we deviated from the pre-registration by using the more powerful one-way ANOVA as opposed to the planned Kruskal–Wallis test. This also means that we deviated from the pre-registered main hypothesis, in the sense that a one-way ANOVA assesses differences in means, rather than the mean rank value, which is assessed in a Kruskal–Wallis test (Field and Hole, 2003).

**FIGURE 1:**
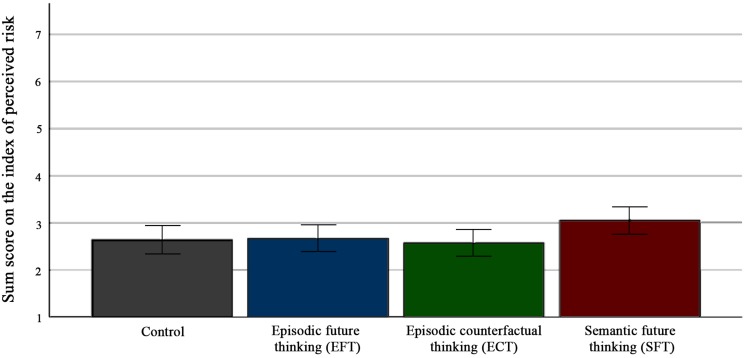
Effect of future thinking on the perceived risk of terror.

Contrary to our prediction, there was no significant difference on the index of perceived risk depending on condition, *F*_3__,__273_ = 2.57, *p* = 0.06, η^2^ = 0.03. The mean scores suggest a trend in the opposite direction of what was predicted in the pre-registered hypothesis, as the mean score on perceived risk was higher in the SFT-condition (*M* = 3.05, *SD* = 1.27) than in the EFT-condition (*M* = 2.67, *SD* = 1.12), the ECT-condition (*M* = 2.58, *SD* = 1.21) and the control condition (*M* = 2.64, *SD* = 1.09). Figure 1 displays the mean scores in the different conditions. However, when excluding six outliers on the index of perceived risk, there was a significant overall difference between the experimental conditions, *F*_3__,__267_ = 2.88, *p* = 0.04, η^2^ = 0.03. As there are no clear grounds for assuming that the outliers were sampled from a different population than the remainder of the scores, the solution with outliers included was kept (Tabachnick and Fidell, 2014). This is also in accordance with the pre-registered plan to retain outliers in all analyses.

#### Exploratory Analyses

In a non-registered, exploratory analysis, we explored whether there was a difference between conditions on the perceived risk of terror using an index consisting only of the items pertaining to the situation that participants were asked to simulate (i.e., a bomb explosion and an armed terror attack). As Levene’s test for equality of variances was significant (*W*_2__,__219_ = 3.48, *p* = 0.03), indicating that the variances in conditions were unequal, Welch’s *F* was used. There was no significant difference on this new index depending on condition, Welch’s *F*_2__,__145__.__31_ = 2.34, *p* = 0.10, η^2^ = 0.02. This did not change when outliers were excluded, Welch’s *F*_3__,__144__.__95_ = 1.47, *p* = 0.23, η^2^ = 0.02.

## Discussion

### Summary

How people perceive the risk of hazards such as terrorism may be an important factor in how they make decisions. It may be that tourists who imagine themselves being caught up in terrorist action at a holiday destination refrain from visiting such destinations since they perceive the risk of such attacks as higher than others who merely think of terrorism in abstract terms. Based on the assumed role of EFT in decision making, and prior research suggesting a relationship between EFT and risk perception, we designed an experiment to assess the impact of future thinking on how people perceive the risk of future terror attacks. Specifically, our research question was centered around the relative influence of engaging in EFT in relation to a future, personal vacation and SFT on the perceived risk of terror attacks.

In a pre-registered hypothesis, we predicted that participants engaging in EFT in relation to a future terror attack while on vacation abroad would perceive the risk of terror attacks as greater than participants asked to think about future terror attacks in an abstract, impersonal way (SFT) participants asked to think about an alternative to a specific episode in their past (episodic counterfactual thinking/ECT) and participants in a passive control condition. This hypothesis was not supported, and the direction of the mean difference is the opposite of what was predicted. Specifically, participants in the EFT-condition did not perceive the risk of terror attacks as higher than participants in the other conditions. These results do not support the idea that whether people engage in EFT or SFT impacts how they perceive the risk of future terror attacks.

Despite a lack of evidence for an effect of future thinking on perceived risk, the analyses of differences on the phenomenological manipulation check measures suggest that participants engaged in the form of thinking which they were instructed to. Regarding temporal orientation, participants in both future thinking-conditions reported that they thought about the future, and participants in the ECT-condition reported that they thought about an alternative to something that happened in the past. Furthermore, participants in both episodic conditions reported more vivid imaginations, and a greater degree of field perspective, than participants in the SFT-condition. As both vividness and field perspective characterize EFT, these results suggest that the experimental manipulations functioned as intended (see McCarroll, 2019, for arguments concerning the importance of observer perspective in EFT). However, it is worth noting that only participants in the ECT-condition, but not in the EFT-condition, reported that they mentally experienced the event to a stronger degree than participants in the SFT-condition. Autonoetic consciousness, enabling a person to pre-experience a future, specific event, is argued to be a chief trademark of mental time travel (Tulving, 1985; McCarroll, 2019), suggesting that participants may not have been engaging in EFT. Despite this, the overall results suggest that participants engaged in the forms of thinking which they were expected to.

As the manipulation checks suggest that the experimental manipulations were effective, the lack of a difference in risk perception between conditions may suggest that perceived risk does not depend on future thinking. Considering that EFT is characterized by vividness and perceived psychological proximity, and that these variables are known to predict an increased risk perception, this would be puzzling (Slovic et al., 1981; Zwickle and Wilson, 2013; Daniel et al., 2015; Bulley et al., 2016; Stein et al., 2018). There are, however, several possible alternative explanations for why we did not find an effect of EFT on the perceived risk of future terror attacks. Importantly, this does not exclude a relationship between EFT and the perceived risk of other hazards.

### Potential Explanations

One could hypothesize that participants in the two episodic conditions did not consider their imaginations realistic, and thus had no reason to perceive terrorism as riskier than participants in the control condition. If an imagined future episode is not perceived as realistic, there is no reason that it would carry weight in how people evaluate hazards. However, judged realism did not correlate with perceived risk, and thus cannot explain the lack of a difference in perceived risk between conditions. This is also true for perceived temporal distance and amount of time spent on the task, neither of which correlated with perceived risk. Another explanation could be that participants did not perceive the imagined events as personally relevant; personal relevance has been identified as an important characteristic of episodic future thoughts (Szpunar, 2010). However, as prior research suggests that perceived risk is associated with travel intentions (Sönmez and Graefe, 1998a, b; Gray and Wilson, 2009), in the sense that a higher perceived risk is associated with weaker intentions, we believe that thinking about future terror attack while abroad would likely be experienced as personally relevant.

One explanation of why EFT did not influence risk perception is that terror attacks are relatively vivid and easy to imagine in the first place. Specifically, if people already have vivid and clear images of how a future terror attack might look like, for example through exposure on news media or through other forms of information, imagining a specific, personal event might not make the event more psychologically proximal or vivid than it already is, and thus not be considered relevant when evaluating the risk of a future terror event on a personal vacation. If this is the case, then episodic future thoughts of hazards may only be relevant for hazards where people do not have very vivid images of the hazard to begin with, so that there is an increase in how easy it is to imagine, and thus also an increase in perceived risk.

In other words: EFT may only increase perceived risk if the psychological distance that is traversed when imagining the event is of sufficient magnitude. Therefore, for events which people do not have vivid scenarios for, such as not previously encountered consequences of climate change like seasonal changes, the perceived risk of hazards associated with such events might be affected by engaging in EFT. Whereas for events which are more vivid, like frequently broadcasted terror attacks, EFT would not further increase the perceived risk. This could be equivalent to the way in which people use vividness in judgments of probability, for example judging the probability of dying from highly imaginable causes of death as greater than the probability of dying from causes of death which are less imaginable but more likely (Lichtenstein et al., 1978). This may explain why EFT seems to increase the perceived risk of environmental challenges (Lee et al., 2018), but did not increase the perceived risk of terrorism in this study.

One other conceivable explanation as to why there was no difference in risk perception between the experimental conditions is that participants focused on different targets while judging risk. If participants in the two episodic conditions focused on their own risk, whereas participants in the control condition focused on the perceived risk for others, participants could have displayed an optimistic bias, perceiving their own risk of being exposed to the hazard as lower than the risk of others being exposed to the same hazard (Weinstein, 1980; Helweg-Larsen and Shepperd, 2001). Thus, even if there was an effect of EFT on risk perception, a difference in focus may have canceled any effect of the experimental manipulation. Although some studies indicate that the optimistic bias might not be present when evaluating the perceived risk of terror attacks (Larsen and Brun, 2011), an optimistic bias could be an explanation of why participants engaging in EFT did not evaluate the risk of terror attacks as greater than participants engaging in SFT or participants in a control condition.

### Methodological Considerations

In addition to two future thinking-conditions and a passive control condition, we opted to include an ECT-condition as an active control condition (De Brigard and Parikh, 2019). However, studies on EFT and ECT suggest that repeated simulations of future thoughts and counterfactuals have differing effects on perceived probability, in the sense that repeated simulations of an imagined future event increase the perceived probability that it will occur, whereas repeated simulations of a past, counterfactual event reduces the perceived probability of the event (De Brigard et al., 2013; Szpunar and Schacter, 2013). One could therefore argue that ECT is ill-suited as an active control condition. However, the methodological alternative would be to use episodic recent thinking, in which participants could not be asked to think about terror attacks, thus introducing the additional confound of a difference in event type (i.e., participants in the EFT- and SFT-conditions would have thought about terrorism, whereas participants in the episodic recent thinking-condition would have thought about another kind of event).

One methodological consideration is the amount of time spent in the experimental conditions. In some other studies (i.e., Lee et al., 2018), the amount of time between conditions has been equalized, which was not the case in the current study. However, it is reasonable to assume that engaging in EFT may be more time intensive than engaging in SFT, suggesting that introducing experimental constraints on the time spent might be artificial. Regardless, the amount of time on the task did not predict perceived risk, suggesting that any difference in the amount of time spent engaging in future thinking or counterfactual thinking did not have implications for perceived risk.

Although our index of the perceived risk of six future terror attacks produced scores with high internal consistency, there is no validation evidence besides face validity, which can be considered insufficient (Meltzoff and Cooper, 2019). However, as participants were instructed that we were interested in their opinions, and they were asked to evaluate the perceived risk of the different forms of future terror attacks, we argue that the answers likely reflect how participants perceive future terror attacks. Self-evidently, developing validated, contextualized measures of relevant future terror attacks to be used to index participants’ perception remains an important goal for future research.

As we deviated from the pre-registration by using a different analysis to test the central hypothesis of the study, it is also relevant to assess whether we had sufficient statistical power to detect a medium difference in a one-way between-subjects ANOVA. According to a *post hoc* power-analysis conducted using G^∗^Power, we had more than sufficient power to detect an effect of medium size (*N* = 277, *d* = 0.5, power = 0.95). This suggests that the lack of a difference most likely cannot be attributed to the total sample size, which increases the explanatory value of the null finding, as it is unlikely that we found no difference because of a lack of statistical power. This lends support to the argument that for the perceived risk of terrorism specifically, there might not be an effect of EFT.

One important additional methodological consideration is the unequal distribution of men and women in the sample, with approximately 77 percent of the sample consisting of women. Some studies suggest gender differences in risk perception (Hicks and Brown, 2013), implying that a sample with a more equal gender distribution might yield different results. However, even if there are gender differences in the level of perceived risk, there is no strong argument for assuming that gender may moderate the relationship between episodic future thinking and risk perception, and taking into consideration that the gender balance amongst students, particularly psychology students (the target population from which we sampled) is unequal (Database for Statistics on Higher Education, 2019), we argue that this does not pose a significant threat to the validity of our results.

### Open Science and Psychology in Tourism

During the last decade there has been an increased attention to the importance of methodological stringency and transparency in medicine, psychology, and other social sciences (Pashler and Harris, 2012; De Boeck and Jeon, 2018). Pre-registering studies with a hypothetico-deductive approach to hypothesis testing is paramount to ensuring that the reliability of the results is not influenced by the use of *questionable research practices*, intentioned or otherwise, and to facilitate the accumulation of scientific knowledge (van’t Veer and Giner-Sorolla, 2016; Wicherts et al., 2016). Pre-registering studies also necessitates a more transparent division between confirmatory and exploratory hypotheses, which makes it easier for readers of scientific literature to evaluate the credibility of the research findings.

The present study is an example of how methodological improvements which are developing rapidly in the field of psychology can be a positive influence on the way studies are conducted in the tourism field more generally. As pre-registered studies are relatively rare in tourism research, we argue that this study not only represents a thematically relevant contribution to furthering the connections between mainstream psychological research and tourism research, but also a methodological contribution to achieving the same goal.

### Future Research and Implications

As previously mentioned, a plausible explanation of why EFT may not be important for how people perceive the risk of future terror attacks is that scenarios of terror attacks are highly vivid, and thus not easily susceptible to any increase in availability as a result of engaging in EFT. One way to test whether the effect of EFT could be moderated by ease of imagination would be to conduct a study using a factorial design with ease of imagination as one factor, and EFT versus SFT as the other factor. This could establish whether there is an interactional effect between ease of imagination and mode of future thinking on risk perception, in the sense that EFT only increases risk perception for scenarios where people initially have difficulty in imagining the consequences. As such, future research on the effect of EFT on risk perception may explore whether the effect is limited to scenarios which people initially have difficulty in imagining.

As highlighted in recent reviews of EFT-measurement (e.g., Miloyan and McFarlane, 2019), there is a shortage of behavioral measures of EFT in the literature. Given that the adaptive significance of future thinking can be directly related to behavior, a reasonable methodological improvement would be the use of relevant, behavioral measures to assess potential behavioral implications of any effect of future thinking on risk perceptions (Suddendorf and Moore, 2011; Miloyan and McFarlane, 2019). Thus, future research could also assess whether EFT can influence behavioral outcomes either through risk perception or other relevant mediators. For example, if future research shows an effect of EFT on perceived risk, this could also have implications for how tourists search for information regarding future destinations, or how they make actual decisions of which destinations to visit and which destinations to avoid.

As mentioned above, assessing behavioral outcomes is a relevant next step using this study as a starting point. If we assume that risk perception is important in explaining travel decisions, a future study could explore whether different forms of future thinking differentially impact travel intentions, or travel decision making. For example, when choosing between different destinations which are perceived as risky, it may matter whether people engage in EFT or SFT for which destination they choose to travel to (Sönmez and Graefe, 1998b). It would be possible to explore the relationship between future thinking and the choice between different risky destinations and assessing whether such a relationship would be mediated by perceived risk. If future thinking impacts perceived risk, which is important in explaining travel decisions, then it is reasonable to assume that this may be a mechanism explaining a potential relationship between future thinking and travel decisions.

Risk perception is assumed to be important in how people make decisions and has been shown to be associated with travel intentions (Sönmez and Graefe, 1998a, b; Gray and Wilson, 2009), in the sense that a greater perceived risk might reduce intentions to travel or increase intentions to stay away. The current results suggest that the way in which people think about the future may not have an impact on perceived risk for terror attacks specifically. Regarding perceived risk, one interpretation of the results is that terrorism as a hazard may be vivid and experienced as psychologically proximal enough so as to not be influenced by imagining specific, personal exposure to terror in the future. Regarding EFT, the results suggest that the significance of this form of future thinking on risk perception may be more context-dependent than previously assumed and may be moderated by how easy it is to imagine the future hazard before engaging in the future thinking. This implies that tourist’s risk perceptions might well be increased by EFT when it comes to other hazards, possibly including the consequences of climate change.

## Conclusion

How people perceive risks is assumed to be important in explaining their decisions, which makes it important to gain further knowledge about the antecedents of risk perception. A pre-registered, experimental study was conducted to explore EFT as such an antecedent. This was based on the idea that imagining a personally relevant, specific future scenario related to terror attacks on a personal vacation might lead to a higher perceived risk of terror, as compared to thinking about the future in an abstract way or thinking about specific episodes that could have occurred in the past. Contrary to our predictions, there was no difference in risk perception between conditions. EFT may still be important for perceived risk, and future research may help in achieving a more complete understanding of which factors predict perceived risk. Such results may be of importance in understanding how people make decisions. Empirical research on EFT and risk perception for events abroad is a starting point for what might be a fruitful interdisciplinary exchange between mainstream psychological research on future thinking and tourism research on how people perceive risk.

## Data Availability Statement

All datasets generated for this study are included in the manuscript/Supplementary Files.

## Ethics Statement

Ethical review and approval was not required for the study on human participants in accordance with the local legislation and institutional requirements. The patients/participants provided their written informed consent to participate in this study.

## Author Contributions

SB and KW conceived of the idea, worked on the research design, critically revised the initial draft of the manuscript, and approved the final version prior to submission. SB gathered the data, conducted the analyses, and wrote the first draft of the manuscript.

## Conflict of Interest

The authors declare that the research was conducted in the absence of any commercial or financial relationships that could be construed as a potential conflict of interest.

## Footnotes

1 https://osf.io/ygptx/?view_only=7861eaaf164646d0a4682d5018c3d383

2 https://osf.io/ygptx/?view_only=7861eaaf164646d0a4682d5018c3d383

## Supplementary Material

DATA SHEET S1 **Data Set**.
